# Corneal Biomechanical Changes after Crosslinking for Progressive Keratoconus with the Corneal Visualization Scheimpflug Technology

**DOI:** 10.1155/2014/579190

**Published:** 2014-09-22

**Authors:** Johannes Steinberg, Toam Katz, Aiham Mousli, Andreas Frings, Maria K. Casagrande, Vasyl Druchkiv, Gisbert Richard, Stephan J. Linke

**Affiliations:** ^1^Department of Ophthalmology, UKE-University Medical Center Hamburg-Eppendorf, Martinistraße 52, 20246 Hamburg, Germany; ^2^Care-Vision Germany, University Medical Center Hamburg-Eppendorf, Martinistraße 52, 20246 Hamburg, Germany

## Abstract

*Purpose.* To evaluate the effect of corneal crosslinking in progressive keratoconus by applying in vivo corneal visualization Scheimpflug technology. *Design.* Longitudinal retrospective study. *Subjects and Controls.* Seventeen eyes of patients treated with corneal crosslinking for progressive keratoconus. *Methods.* Corneal visualization Scheimpflug technology analyses (research software version 6.07r08) of subjects with progressive keratoconus before and 3 months after corneal crosslinking (CXL) were reviewed retrospectively. *t-*test (for normal distribution) and Wilcoxon matched-pairs test (if not normally distributed) were used to test for statistically significant differences between pre- and post-CXL analyses. *Results.* We demonstrated statistically significant differences for the intraocular pressure (median: +3 mmHg, *P* = 0.004), the central corneal pachymetry (pachy; mean: −35 *µ*m, *P* < 0.001), the timespan between the air impulse release and the first applanation of the cornea (A1time; median: +0.12 ms, *P* < 0.05), and the timespan between the air impulse release and the second applanation of the cornea (A2time; median: −37 ms, *P* < 0.05). *Conclusions.* With the A1time and the A2time, we identified two parameters that demonstrated a statistically significant improvement of the biomechanical properties of the cornea after CXL. Despite the known initial decrease of the pachymetry after CXL, none of the analyzed parameters indicated a progression of the keratoconus.

## 1. Introduction

Keratoconus (KC) is a bilateral noninflammatory disease of the cornea characterized by progressive corneal thinning and ectasia [1]. Introduced in 2003, corneal crosslinking (CXL) was the first treatment aimed at the pathogenetic cause of KC by potentially changing the intrinsic biomechanical properties of the corneal collagen [2]. Histologically, CXL causes an increase of the fiber diameter and chemical bonding between corneal microstructural components, leading to a higher mechanical stiffness of the cornea [3, 4]. A reduced susceptibility to enzymatic degradations has also been described [5, 6].

Over the past few years, long-term studies concentrating on in vivo topography and tomographic analyses have been published to demonstrate that CXL is an effective treatment for stopping the natural course of progressive KC [7–9].

The next step in extending our knowledge of CXL, and thereby in optimizing treatment, is to gain a better understanding of not only the morphological but also the in vivo biomechanical effects of CXL. We could then make a better evaluation of the different CXL strategies, which may possibly enable individualized therapy strategies.

The aim of the current study was to analyze biomechanical changes of the cornea after CXL for progressive KC by applying in vivo corneal visualization Scheimpflug technology (CST) combined with new research software (version 6.07r08).

## 2. Materials and Methods

### 2.1. Setting

This retrospective study was performed in cooperation between the Department of Ophthalmology, University Medical Center Hamburg-Eppendorf, and the Care Vision Eye Clinic Hamburg.

### 2.2. Participants

Patients received corneal crosslinking (CXL) for progressive keratoconus.

Inclusion criteria for CXL were

- diagnosis of a progressive keratoconus that is pentacam topography documented progression of maximum *K*-values > 0.5 D within 6 months. The diagnosis of KC was confirmed by asymmetry of the corneal surface (KISA%) of >100 [11]. Further, all patients were classified as stage 1 or 2 according to the Amsler-Krumeich classification [12];
- best corrected visual accuracy of at least 0.4 logMAR;
- absence of corneal scars;
- signed consent form including the information of the off-label status of the therapy.

Exclusion criteria for CXL were

- central corneal thickness < 400 microns (even after intraoperative corneal swelling with a hypotonic solution);
- pregnancy;
- severe dry eye syndrome;
- inflammation of the ocular surface, the anterior chamber or the eyelids.

None of the included eyes received surgical procedure before the CXL.

### 2.3. Data Collection

Biomechanical analyses were performed with the Corvis ST (CST; Oculus Inc., Dudenhofen, Germany). After using the automated export function of the CST, the data were recalculated by applying the CST research software version 6.07r08 developed by Oculus. This recalculation process adds additional parameters to the analyses. Moreover, it provides extra quality scores that help in further assessing the value of the data. The CST and the analyzed parameters of the original software have been explained elsewhere [13].

The new research software adds the following parameters.

**Figure 1:**
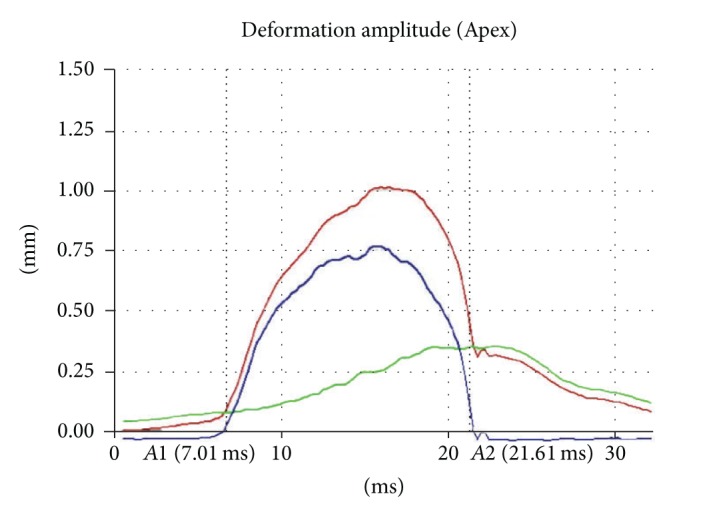
Deformation/deflection/eye movement diagram. Blue line: deflection; red line: deformation; green line: whole eye movement.

**Figure 2:**
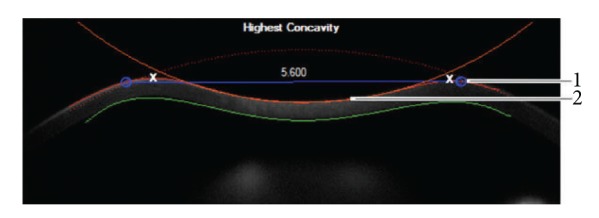
Display of the deflection length and the highest concavity. (1) Deflection length; (2) highest concavity.

- Deflection amplitude [mm] (Figure 1): displacement of the corneal apex in reference to the overlaid cornea in initial state (blue line). The movement of the corneal apex is compensated by the whole eye movement. Only the movement of the cornea is described by this parameter. The red line in Figure 1 displays the deformation amplitude (DA). The DA is determined by the deformation of the corneal apex in vertical direction. It is the sum of deflection amplitude and whole eye movement (green line).
- Deflection length [mm] (Figure 2): length of a line (blue line, Figure 2) that describes the deflected part of the cornea compared to the undeformed cornea in the initial state (red dotted line). The two end points (blue circles) are fitted to the positions where the shape of the outskirts of the cornea does not differ from the cornea in the initial state. This allows more robust information to be obtained on the applanated part of the cornea at the time of the first and second applanation compared to just the applanation length.
- Radius (3P) [mm]: radius of curvature at maximum deformation, 3-point-fit.
- A1 deflection length [mm]: deflection length at the time of the first applanation.
- HC deflection length [mm]: deflection length at the time of the highest concavity.
- A2 deflection length [mm]: deflection length at the time of the second applanation.
- HC deflection amp. [mm]: deflection amplitude of the highest concavity.
- A1 deflection amp. [mm]: deflection amplitude of the first applanation.
- A2 deflection amp. [mm]: deflection amplitude of the second applanation.
- Deflection amp. max. [mm]: maximum deflection amplitude.
- Deflection amp. max. [ms]: time of the maximum deflection amplitude.

The CST analyses were performed with a median of 0 days before and 84 days (3 month) after CXL. The exact time intervals for every patient are displayed in Table 1.

The CST displays a quality specification grade (QS) based on the patient's alignment and the integrity of the data record for every analysis. Only CST analyses with a status “OK” for all available QS were included in the statistical analyses. If the patient had used contact lenses, a minimum of 14 days (hard lenses) or 4 days (soft lenses) of contact lens abstinence was maintained. To avoid a potential bias attributed to diurnal variations of the corneal thickness and the anterior and posterior corneal surface, both the pre-CXL and the 3-month post-CXL analyses were performed between 8 and 10 a.m. [14]. Only one eye of every subject was included in the statistical analyses.

### 2.4. Surgical Technique

Standard corneal CXL was conducted using Dresden protocol as previously reported [2]. According to that, CXL was conducted under sterile conditions in the operating room. Oxybuprocaine 0.4% eye drops were applied for preoperative local anesthesia. After inserting the eyelid speculum, the central 9 mm of the corneal epithelium was cautiously removed using 20% alcohol solution (20 seconds) and a blunt knife.

After de-epithelialization, an ultrasound-pachymetry was performed to ensure a central corneal thickness >400 *µ*m. In case of a central corneal thickness >400 *µ*m, a photosensitizer, riboflavin 0.1% solution (10 mg riboflavin-5-phosphate in 10 mL dextran-T-500 20% solution) was applied every 2 minutes for 30 minutes. If the pachymetry was <400 *µ*m, we applied 0.1% hypo-osmolar riboflavin solution. After 30 minutes, the application of riboflavin was followed by another pachymetry to confirm a central corneal thickness of at least 400 *µ*m. If the corneal thickness was thinner then 400 *µ*m, we continued the application of the hypotonic solution until the pachymetry exceeded 400 *µ*m and continued with the hypotonic solution throughout the irradiation. If the central pachymetry was >400 *µ*m in both measurements, we applied the riboflavin 0.1% solution as described above. The application of riboflavin was continued every 5 minutes during the following irradiation.

The UVA irradiation was started using UV diodes (370 nm; Peschke Lasertechnik, Waldshut-Tiengen, Germany) with the UVA light focusing on the cornea while protecting the limbus. Before each treatment, the desired irradiance of 3 mW/cm^2^ was controlled with a UVA meter (LaserMate-Q; LASER 2000, Wessling, Germany) and, if necessary, regulated with the potentiometer. The patient's cornea was irradiated with the UVA-light diodes for 30 minutes. Treated eyes were dressed with a soft contact lens bandage for 4 days and were medicated with antibiotics (ofloxacin drops 4 times/day), nonsteroidal anti-inflammatory drugs (diclofenac drops 4 times/day), and lubricants (phospholipidic microemulsion drops tapered 8 times/day). All eye drops were free of preservatives. After CL removal on 4th postoperative day the therapeutic regimen was changed to fluorometholone 0.2% drops (4 times/day) and lubricants (phospholipidic microemulsion drops, 8 times/day). During the 5th and 10th week after CXL, fluorometholone was reduced by 1 eye drop every two weeks.

Our study adhered to the tenets of the Declaration of Helsinki. Informed consent for retrospective data analysis and approval of the Institutional Review Board (IRB)/Ethics Committee for the study were obtained.

### 2.5. Statistical Analysis

Before statistical analyses, all CST parameters were automatically exported into a spreadsheet program (Microsoft Office Excel) using the original software of the device. The data were recalculated using the new research software of the CST developed by Oculus (v.6.07r08). For statistical analyses, the general purpose statistical software (STATA version 11.0; StataCorp) was applied. For normal distributions, a *t*-test was used to test for statistically significant differences between pre- and postoperative analyses. If a parameter was not normally distributed, a Wilcoxon matched-pairs test was used. A *P* value less than 0.05 was considered statistically significant.

## 3. Results

Our database contained records of 22 patients with progressive KC who received CST analyses before and after CXL. Of these, 17 eyes of 17 patients (2 females, 15 males; mean age 27 years; min/max: 20/47 years) displayed CST analyses with high quality results before and median 3 months after CXL. Descriptives are displayed in Table 1.

More than 75% of the examinations were conducted only minutes before the treatment and mean followup was 3 months. High values of the Belin Ambroso Index (BAD_D), the topographic astigmatism and/or the maximum keratometry of the corneal surface (*K*max) could be demonstrated for every patient before the treatment.

Table 2 displays the changes of the exported CST parameters before and 3 months after CXL.

Of the 24 automatically exported parameters of the CST, 4 parameters displayed statistically significant differences between pre- and postoperative examination: the intraocular pressure (IOP; median: +3 mmHg), the central corneal pachymetry (pachy; mean: −35 *μ*m), the timespan between the air impulse release and the first applanation of the cornea (A1time; median: +0.12 ms), and the timespan between the air impulse release and the second applanation of the cornea, which occurs when the cornea passed the point of maximum impression and is on its way back to the initial state (A2time; median: −37 ms) (see also Table 3).

All these statistically significant changes indicate an increase of the corneal stiffness after the treatment. Even considering the parameters without statistically significant changes, none of the analyzed parameters demonstrated an alteration, which might suggest a worsening of the corneal biomechanical properties after CXL. The differences of the parameters with statistically significant changes are outlined in Table 3.

We calculated the sample sizes for the new deflection parameters based on our data (Table 4).

Based on the distribution and the number of subjects included (see also *N* in Table 2) the required sample sizes vary between 21 and >10,000. It should be born in mind that the calculated sample size also strongly depends on the follow-up period. An extended followup might reduce the calculated sample size distinctively.

## 4. Discussion

We identified statistically significant differences between four parameters obtained before and 3 months after CXL: IOP, central corneal pachymetry, “A1time,” and “A2time.”

Over the past few years, several methods for measuring the geometric structure of the cornea have been used for analyzing corneal geometrics and to describe corneal pathologies. These devices have improved our understanding of corneal pathologies and helped us to identify pathologies like KC in its early stages. Besides these improvements in structural analyses, in vivo analyses of biomechanical changes have been developed. The first device allowing in vivo analyses of the cornea was the Ocular Response Analyzer (ORA) (Reichert Technologies) [15, 16]. Only a few study groups have analyzed in vivo biomechanical changes in progressive KC after CXL [17–20]. Unfortunately, ORA analyses of post-CXL changes have not yielded consistent findings [17, 18, 20, 21].

Recently, Oculus introduced a new device for in vivo analyses of corneal biomechanics called “Corneal Visualization Scheimpflug Technology” (CST). Like the ORA, it uses a precise collimated air pulse to cause the cornea to move inwards. However, in contrast to the ORA, it uses high-speed Scheimpflug technology to follow the movement of the cornea throughout the whole dynamic process of inward and outwards motion. In this way a range of parameters are generated which enable complex analyses of the viscoelastic properties of the cornea. Until now, only a few studies comparing biomechanical properties of KC and normal eyes have been published [13, 22].

Tian et al., using the CST software version 1.00r30, found the deflection amplitude (DA) to be the best parameter for differentiating between KC and normal eyes (NE) by demonstrating a higher DA in KC (their “DA” is equivalent with our “defampmax (mm)”) [13]. They also demonstrated a lower concavity curvature and faster corneal applanation velocity in KC compared to NE. Ali et al., comparing data of 45 KC eyes and 103 NE, also found the DA to be the potentially strongest parameter for differentiating between KC and NE, with DA being higher in KC (KC: 1.37 ± 0.21 mm; NE: 1.05 ± 0.11 mm, *P* < 0.001). However, because of the only minor ROC (receiver operating characteristic) areas with no ideal cut-off values, they concluded that the DA may be a useful adjunct in KC assessment and monitoring but cannot solely discriminate between normal and keratoconic corneas.

Tomita et al. used the CST for analyzing biomechanical changes of the cornea after CXL for progressive KC [19]. They compared the results of accelerated versus conventional CXL in progressive KC by applying ORA and CST with a follow-up of 1 year. Similar to our study, they included subjects with KC classified as first or second stage according to the Amlser-Krumeich classification [12]. Unfortunately, they did not give information about the criteria of KC progression leading to the indication for CXL. As in previous studies, they could not demonstrate statistically significant changes after conventional CXL using the ORA for in vivo biomechanical analyses. However, using CST analyses, they found changes in three parameters: the deformation amplitude (DA; −0.02 mm), the distance between corneal bending points (“peak distance”; +0.42 mm) and the radius of the curvature at the time of highest concavity of the cornea (+0.1 mm). Although these changes indicate a higher stiffness of the cornea, the changes in these parameters were not statistically significant at 1 year after conventional CXL.

We demonstrated statistically significant changes in the A1time (median: +0.12 ms) and the A2time (median: −0.37 ms). In addition, the IOP (median: +3 mmHg) and the central corneal pachymetry (pachy; mean: −35 *μ*m) demonstrated statistically significant changes. In line with previously published data, the DA decreased slightly, but not on a statistically significant level (−0.9 mm, *P* = 0.155). Also the peak distance and the radius at the time of the highest concavity changed with the same direction as that demonstrated by Tomami et al., but again did not reach a statically significant level (peak distance: −0.49 mm, *P* = 0.165; radius: +0.17 mm; *P* = 0.488).

The changes of “A1time” and “A2time” at 3 month after CXL indicate an increase in the corneal resistance after the treatment. Because of the increased “stiffness” of the corneal tissue, the time until the cornea reaches the status of the first applanation (A1time) increased and the time of the second applanation (after the cornea passed the point of maximum deformation [A2time]) decreased. Correspondingly, Tian et al. demonstrated a statistically significant longer “A1time” and a shorter “A2time” in NE than in KC on analyzing the biomechanical properties of the cornea in KC and NE with the CST [13].

Huseynova et al. showed that the biomechanical analyses measured with the CST are strongly influenced by the IOP and the pachymetry [23]. This is of curial importance, especially in cross-sectional-analyses. In longitudinal studies, the IOP or the pachymetry should not matched or accounted before statistical analyses to avoid generating confounding factors. An increase in pachymetry leads to an increase in the measured IOP values since more pressure is needed to applanate the cornea regardless of the real IOP [24, 25]. We demonstrated a statistically significant increase in the IOP despite a decrease of the pachymetry. This relationship can be seen as a further biomechanical indication of an increase of corneal stiffness. Accordingly, Tian et al., analyzing KC and NE, demonstrated a statistically significantly lower IOP in their KC group [13]. The initial decrease of corneal pachymetry after CXL has been described in several studies [26–28]. These studies also demonstrated an increase in the pachymetry over the months following treatment. Because pre- and post-CXL measurements were performed between 8 and 10 a.m., the diurnal change of IOP should be negligible [29, 30]. Several study groups have already demonstrated a high repeatability and accuracy of IOP and pachymetry measurement with the CST [23, 31, 32].

We used the new research software version 6.07r08 for our analyses. This software additionally provides new deflection parameters. Focusing on deformation-related parameters, as in the mentioned above, the sum of the deflection (real corneal apex movement) and the whole eye movement are analyzed. As shown in the methods section, the deflection describes the displacement of the corneal apex in reference to the overlaid cornea in its initial state. Therefore, the movement of the corneal apex is compensated by the whole eye movement and only the “real” movement of the cornea is described.

Unfortunately, despite applying the new test software, we could not demonstrate further statistically significant differences in the deflection-based parameters. This is probably related to the small sample size and the short follow-up of our study. Different study groups have demonstrated ongoing changes of the cornea even years after the CXL [7–9].

The strength of our study is the methodological standardization: We only implemented CST analyses with objectively measured high-quality standards (included QS “OK” or “Model deviation”; excluding all analyses with an “Alignment” or “Lost images” warning). Excluding confounding factors such as wrong alignment is crucial because the CST measures corneal changes caused by a precise collimated air pulse. If the alignment is off-center, the analysis is severely compromised. In addition, 76% of the included pre-CXL analyses were performed only minutes before the CXL, reducing the probability of potential changes between the last examination before the CXL and the time of the treatment. Further, all analyses were performed between 8 and 10 a.m. to reduce diurnal variations. We were able to apply the latest CST research software. As already mentioned, besides an improved feedback regarding the quality of the analyses (additional QS scores), this version provides (deflection-based) parameters that are not compromised by back and forth movements of the eye that occur as a reaction to the air impulse during the measurement.

We acknowledge the fact that our study has certain limitations. We only included 17 eyes in our retrospective analyses. Additionally, 24% of the pre-CXL analyses were not performed immediately before CXL but within 6 weeks before the treatment (range 16–44 days before CXL). This might affect the results due to potentially unknown biomechanical changes between the pre-CXL measurement and the treatment. Therefore, a prospective study design with requested CST on the same day as CXL would be helpful to minimize the time interval between analysis and treatment. Alike, the time range of the post-CXL examinations might bias the results. As displayed in Table 1, 89% of the examinations post-CXL were conducted 70–90 days after the treatment. One patient was examined 56, and another patient 98 days after CXL. These outliers potentially affect the results by either not detecting subtle changes due to a too short timespan after CXL, or overrating changes in the context of our median 3 month follow up. Unfortunately, we could not find other prospective or retrospective studies analyzing changes after CXL for progressive KC which report the time range of their follow up intervals pre- and post CXL. Therefore, further comparisons are impossible [7, 33–35]. Nevertheless, because of a high percentage of examinations conducted only few minutes before the treatment (76% of the included examinations) and almost 90% of the examinations performed within a time range of 20 days at the 3 month follow up, we think that our results contribute to the understanding of biomechanical changes after CXL for progressive KC.

## 5. Conclusions

We identified two parameters, A1time' and A2time, that indicate an improvement of the corneal biomechanical properties after corneal CXL for progressive KC. None of the other CST parameters revealed statistical significant changes, demonstrating at least a stabilizing effect of CXL on corneal biomechanics. However, studies with a longer followup and larger sample sizes are warranted.

## Figures and Tables

**Table 1:** Descriptives.

Patient	Sex	Age	Days before CXL	Days after CXL	BAD_D	Pachy min (*μ*m)	Astig. (D)	*K*max (D)
1	Male	21	0	98	7.18	453	4.20	51.66
2	Female	28	0	84	7.06	489	2.50	54.79
3	Female	47	−21	56	4.42	464	3.00	49.74
4	Male	27	0	91	7.62	462	4.70	50.59
5	Male	20	0	70	7.84	454	7.80	58.00
6	Male	39	0	77	14.07	520	2.00	60.05
7	Male	24	0	84	9.30	480	5.90	59.70
8	Male	29	−23	70	10.76	450	6.10	59.25
9	Male	20	0	91	5.56	508	4.70	54.28
10	Male	37	0	91	8.22	501	2.20	59.99
11	Male	21	−16	91	6.19	522	4.20	47.48
12	Male	43	0	84	0.82	545	6.30	49.46
13	Male	27	0	91	9.27	462	5.70	57.00
14	Male	29	0	77	12.11	454	6.90	62.57
15	Male	47	0	91	8.19	451	6.40	54.09
16	Male	46	0	70	0.67	571	6.80	49.53
17	Male	25	−44	84	3.30	475	2.20	46.75

Days before CXL and days after CXL: time interval between Corvis ST analysis and corneal crosslinking; BAD_D: Belin-Ambrosio display-enhanced ectasia total deviation value. [10]; pachy min: corneal thickness at the thinnest point; Astig: topometric astigmatism front surface; *K*max_: steepest keratometry of the front surface.

**Table 2:** CST parameter before and 3months after CXL.

Parameter (unit)	*N*	Before CXL			*N*	3 months after CXL			P*
		Min/Max	Median (Q25/Q75)	Mean (±SD)		Min/Max	Median (Q25/Q75)	Mean (±SD)	
IOP (mmHg)	17	7.50/17.00	11.00 (9.00/12.50)	11.26 (2.76)	17	8.00/45.50	14.00 (12.00/16.00)	15.12 (8.31)	**0.004** ^†^
pachy (*µ*m)	17	415/562	487 (473/539)	498 (42.99)	17	365 /548	452 (438 /502)	463 (45.33)	**<0.001**
defampmax (mm)	17	0.94/1.41	1.18 (1.08/1.26)	1.16 (0.13)	17	0.44/1.51	1.09 (1.04/1.21)	1.10 (0.22)	0.155^†^
A1time (ms)	17	6.49/7.50	6.93 (6.78/7.13)	6.96 (0.28)	17	6.61/10.68	7.12 (6.87/7.35)	7.29 (0.92)	**0.024** ^†^
A1length (mm)	17	1.33/1.89	1.70 (1.53/1.79)	1.65 (0.18)	17	1.22/1.86	1.69 (1.60/1.76)	1.64 (0.18)	0.891
A1velocity (ms)	17	0.09/0.22	0.14 (0.13/0.17)	0.15 (0.04)	17	0.03/0.22	0.15 (0.13/0.17)	0.15 (0.05)	0.902
A2time (ms)	17	21.41/22.86	22.25 (21.84/22.46)	22.16 (0.46)	16	19.07/22.93	21.65 (21.54/21.95)	21.66 (0.83)	**0.039** ^†^
A2length (mm)	17	0.80/2.41	1.27 (1.09/1.87)	1.43 (0.47)	17	0.78/2.12	1.55 (1.08/1.82)	1.49 (0.44)	0.702
A2velocity (ms)	17	−0.67/−0.26	−0.46 (−0.52/−0.39)	−0.45 (0.11)	16	−0.57/−0.24	−0.44 (−0.49/−0.34)	−0.42 (0.10)	0.348
hctime (ms)	17	16.17/18.25	17.09 (17.09/17.56)	17.27 (0.50)	17	15.25/18.71	17.09 (16.63/17.56)	17.04 (0.77)	0.292
peakdist (mm)	17	2.14/5.62	4.81 (4.68/5.10)	4.46 (1.07)	17	2.01/5.92	4.58 (2.49/5.08)	3.97 (1.36)	0.165
radius (mm)	17	4.27/9.48	5.80 (5.25/6.29)	6.04 (1.27)	17	4.52/9.96	6.04 (5.25/6.56)	6.21 (1.41)	0.488
radius3p (mm)	17	4.27/9.48	5.96 (5.30/6.35)	6.12 (1.28)	17	4.52/9.96	6.04 (5.25/6.56)	6.23 (1.43)	0.683
A1deformationamp (mm)	11	0.09/0.32	0.12 (0.10/0.15)	0.14 (0.06)	8	0.07/0.22	0.13 (0.11/0.18)	0.14 (0.05)	0.544
hcdeformationamp (mm)	11	0.99/1.55	1.14 (1.05/1.33)	1.19 (0.18)	8	0.95/1.41	1.14 (1.07/1.25)	1.16 (0.15)	0.838
A2deformationamp (mm)	11	0.18/0.53	0.38 (0.33/0.42)	0.37 (0.09)	7	0.21/0.44	0.38 (0.28/0.43)	0.36 (0.08)	0.270^†^
A1deflectionlength (mm)	11	1.85/3.15	2.30 (2.02/2.46)	2.32 (0.36)	7	1.55/2.89	2.33 (2.06/2.79)	2.38 (0.48)	0.612^†^
hcdeflectionlength (mm)	11	5.50/6.12	5.80 (5.75/6.03)	5.85 (0.19)	8	5.70/6.16	5.90 (5.78/5.96)	5.89 (0.15)	0.699
A2deflectionlength (mm)	10	1.90/3.63	3.00 (2.51/3.09)	2.85 (0.48)	5	2.05/3.50	3.12 (3.04/3.31)	3.00 (0.56)	0.095^†^
hcdeflectionamp (mm)	11	0.81/1.36	0.97 (0.89/1.09)	1.01 (0.17)	11	0.75/1.36	0.97 (0.91/1.09)	1.00 (0.17)	0.592^†^
A1deflectionamp (mm)	11	0.07/0.31	0.10 (0.07/0.11)	0.11 (0.07)	11	0.04/0.47	0.10 (0.07/0.18)	0.14 (0.12)	0.188
A2deflectionamp (mm)	11	0.03/0.33	0.13 (0.06/0.19)	0.14 (0.09)	11	0.00/0.69	0.15 (0.09/0.19)	0.20 (0.20)	0.247
deflectionampmax (mm)	11	0.81/1.40	0.98 (0.92/1.09)	1.02 (0.17)	8	0.76/1.26	1.00 (0.93/1.13)	1.02 (0.16)	0.885^†^
deflectionampmax (ms)	11	15.99/16.83	16.40 (16.17/16.74)	16.44 (0.31)	8	14.35/17.20	15.99 (15.89/16.38)	16.01 (0.81)	0.254

*P* values reaching statistical significance are in bold font.

IOP: intraocular pressure; pachy: central corneal pachymetry; defampmax: maximum corneal deformation amplitude; A1time: time of the first applanation; A1length: Length of the first applanation; A1velocity: velocity of the corneal apex at the first applanation; A2time: time of the second applanation; A2length: length of the second applanation; A2velocity: velocity of the corneal apex at the second applanation; hctime: highest concavity, time of the maximum deformation; peakdist: distance between both non-deformed peaks; radius: radius of curvature at maximum deformation, calculated with “parabolic fit”; radius3p: radius of curvature at maximum deformation, 3-point-fit; A1deformationamp: deformation amplitude at the time of the first applanation; hcdeformationamp: deformation amplitude at the time of the highest concavity; A2deformationamp: deformation amplitude at the time of the second applanation; A1deflectionlength: deflection length at the time of the first applanation; hcdeflectionlength: deflection length at the time of the highest concavity; A2deflectionlength: deflection length at the time of the second applanation; hcdeflectionamp: deflection amplitude of the highest concavity; A1deflectionamp: deflection amplitude of the first applanation; A2deflectionamp: deflection amplitude of the second applanation; deflectionampmax (mm): maximum deflection amplitude; deflectionampmax (ms): time of the maximum deflection amplitude.

∗Paired *t*-test.

^†^Differences are not normally distributed. Therefore, Wilcoxon matched-pairs test was used.

**Table 3:** Differences for the parameters with statistically significant changes after CXL.

Parameter (unit)	Before CXL		3 months after CXL		Differences	
	Median (Q25/Q75)	Mean (±SD)	Median (Q25/Q75)	Mean (±SD)	Median (Q25/Q75)	Mean (±SD)
IOP (mmHg)^†^	11.00 (9.00/12.50)	11.26 (2.76)	14.00 (12.00/16.00)	15.12 (8.31)	**+3.00 (+4.50/0.00)**	+3.85 (7.33)
pachy (*µ*m)∗	487 (473 /539)	498 (42.99)	452 (438 /502)	463 (45.33)	−41 (−14 /−50)	**−35.00 (26.05)**
A1time (ms)^†^	6.93 (6.78/7.13)	6.96 (0.28)	7.12 (6.87/7.35)	7.29 (0.92)	**+0.12 (+0.32/0.00)**	+0.33 (0.80)
A2time (ms)^†^	22.25 (21.84/22.46)	22.16 (0.46)	21.65 (21.54/21.95)	21.66 (0.83)	**−0.37 (+0.10/−0.79)**	−0.47 (0.78)

*Normally distributed.

^†^Not normally distributed.

The relevant differences are in bold font.

**Table 4:** Sample size calculation for the deflection-based parameters calculated using the new CST research software.

Parameter	Delta (Mean/SD)	Required sample size∗
A1deflectionlength (mm)	−0.08 (0.53)	347
hcdeflectionlength (mm)	−0.03 (0.20)	350
A2deflectionlength (mm)	−0.26 (0.40)	21
hcdeflectionamp (mm)	0.003 (0.11)	10555
A1deflectionamp (mm)	−0.026 (0.06)	44
A2deflectionamp (mm)	−0.06 (0.16)	58
deflectionampmax (mm)	−0.018 (0.123)	368
deflectionampmax (ms)	0.43 (0.98)	43

*Based on our data (*n* = 17, 3-month followup), this sample size is required to prove the differences with the *t*-test at the significance level of 0.05 and a test power of 80%.
